# Acellular dermal matrix in skin wound healing in rabbits - histological and histomorphometric analyses

**DOI:** 10.6061/clinics/2021/e2066

**Published:** 2021-03-01

**Authors:** José da Conceição Carvalho-Júnior, Fabiana Zanata, Antônio Carlos Aloise, Lydia Masako Ferreira

**Affiliations:** Divisao de Cirurgia Plastica e Reconstrutiva, Universidade Federal de Sao Paulo, Sao Paulo, SP, BR

**Keywords:** Wound Healing, Rabbit, Dermal Matrix

## Abstract

**OBJECTIVES:**

To analyze the histology and histomorphometry of healing associated with acellular dermal matrix in skin wounds in rabbits.

**METHODS:**

Twelve male rabbits were divided into two groups: the control group (CG) and the matrix group (MG). Three skin wounds with a total area of 20 × 20 mm were created on the dorsal region of each animal. Photographic records of the lesions taken over a 21-day period and use of the ImageJ program allowed calculation of the wound contraction rate. The lesions were biopsied on days 3, 14 and 21 for histomorphometric analysis to define the thicknesses of the dermis and epidermis (hematoxylin-eosin) and calculate the densities of type I and type III collagen (picrosirius).

**RESULTS:**

No significant difference in the healing rate was found between the groups (*p*>0.05). The MG presented greater epidermal thickness on day 3 (*p*<0.05) and on days 14 and 21 (*p*<0.001). The MG presented greater dermal thickness throughout the study period (*p*<0.05). The type I collagen density was higher in the MG throughout the study period (*p*<0.05), and the type III collagen density was higher in the MG on days 3 and 14 (*p*<0.05) and on day 21 (*p*<0.001).

**CONCLUSION:**

The use of acellular dermal matrix increased the thickness of the dermal and epidermal layers and the amount of type I and III collagen during skin wound healing and did not alter the rate of wound contraction.

## INTRODUCTION

Whole and healthy skin is critical for maintaining homeostasis. It plays an important role in protecting against infections, in thermal regulation and in water balance. Skin lesions affect all of these functions; therefore, rapid and effective healing is essential for the body (1-3).

Several clinical conditions are characterized by wounds with full skin thickness loss in which the dermis and epidermis are completely destroyed. Unlike the epidermis, the dermal layer is unable to regenerate and is replaced by scar tissue devoid of the characteristics necessary for adequate aesthetic and functional recovery (4).

Conventional surgical treatments with autogenous or autologous grafts are able to replace the skin in damaged areas but result in a new wound at the patient’s donor site. Another alternative is the use of allografts, which, despite the benefits of the initial coverage, such as reduced water loss, pain and infection risk, can result in rejection due to antibody-antigen complexes (5,6).

Extensive skin defects caused by trauma, thermal injury or tumor resection that are not susceptible to primary closure remain a challenge in reconstructive surgery, especially when there is insufficient donor skin. An alternative approach to the use of conventional skin grafts is the use of tissue engineering, which employs the triad of cells, scaffold and growth factors to promote tissue regeneration (7).

Acellular dermal matrix (ADM) is a fully artificial acellular biomaterial composed of two layers of that can be used as a scaffold in skin tissue engineering. The upper layer consists of a thin silicone layer, which, like the epidermis, is effective in controlling water loss and preventing bacterial invasion. The lower layer comprises a highly porous mesh composed of bovine collagen and chondroitin-6-sulfate derived from shark cartilage (8,9).

In recent years, ADM has been widely used in complex cases, such as for burns (10), tympanic membrane perforations (11), diaphragmatic defects (12,13), chronic diabetic foot wounds (14), trauma to the extremities (15) and breast reconstruction (16).

ADM has been considered a good dermal substitute because it reproduces many skin characteristics, including elasticity, resistance and barrier functions against microorganisms in the wound bed (17). However, the clinical benefit of matrices has not been fully elucidated due to evidence that low vascularization in the recipient bed can limit the supply of nutrients, immune cells and oxygen to the wound site (18,19). These factors can lead to low regenerative capacity, high infection rates, foreign body reactions and hypertrophic scar formation (20).

In this context, because vascularization is the main factor associated with the problems presented by the clinical use of matrices, histological and histomorphometric analyses of the healing of skin wounds covered with ADM were performed in an area without a compromised vascular bed.

## METHODS

### Ethical issues

This study was approved by the Research Ethics Committee and the Animal Use Ethics Committee of Federal University of São Paulo under number 9824290116. This study conformed to The ARRIVE Guidelines Checklist for studies on animals as well as the criteria of the Brazilian College of Animal Experimentation (Colégio Brasileiro de Experimentação Animal - COBEA).

### Animals

Twelve adult male New Zealand rabbits (*Oryctolagus cuniculus*) with a mean weight of 3.0 (2.8-3.2) kg were acquired from the Center for the Development of Experimental Models of UNIFESP. Randomization was performed in blocks of two using the website http://www.randomization.com. The animals were placed in individual cages in a controlled temperature environment (18 to 20°C) and were fed *ad libitum*, according to the specifications of the Animal Use Ethics Committee of UNIFESP.

### Groups

The rabbits were divided into two groups:

-  Control group (CG) - second-intention wound healing; and
-  ADM group (MG) - ADM wound coverage.

### Surgical procedure

For all surgical procedures, the animals were placed on a surgical bench for the induction of general anesthesia via the intramuscular administration (gluteal region) of a solution of 80% ketamine (40 mg/kg) and 20% xylazine (5 mg/kg).

**Figure 1:**
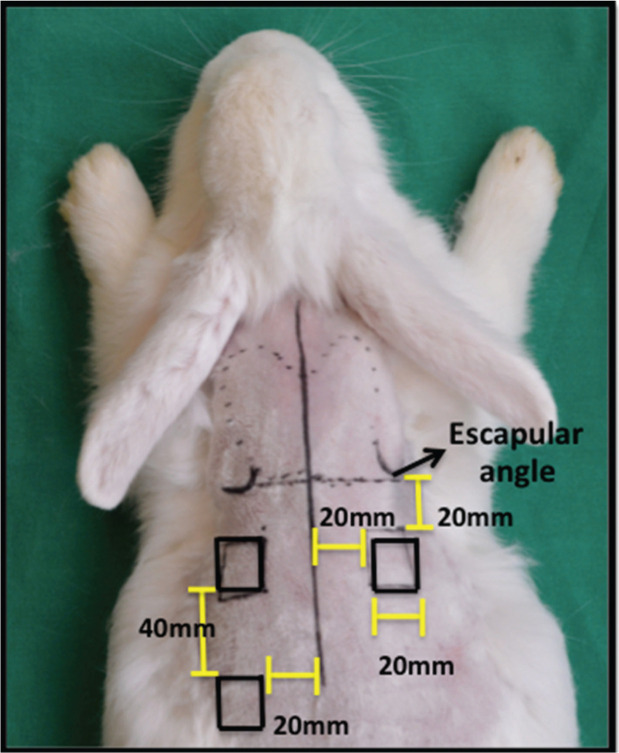
Points of reference for making the wounds.

After shaving the fur on the dorsum with a Philips QG3339 electric trimmer, three full-thickness wounds were made on the skin of the animals according to the method described by Cross et al. A 20 × 20 mm^2^ metal plate was used as a standard template to define the lesions, which were marked with a 500 Texta Extra Fine PR8538 marker. Two lesions were made on the dorsal region, 20 mm below the scapular angle and 20 mm from the median sagittal line. The third lesion was made 40 mm from the lower margin of the cranial lesion to the animal’s left and 20 mm from the dorsal sagittal line (Figure 1). Sterile surgical drapes were placed following antisepsis with 2% chlorhexidine-alcohol. Next, local anesthesia was performed with 2% lidocaine in the subdermal plane throughout the incision site, and three square wounds measuring 20 × 20 mm^2^ were made by excision of the full skin thickness to the subcutaneous plane with a number 15 scalpel, followed by hemostasis.

**Figure 2:**
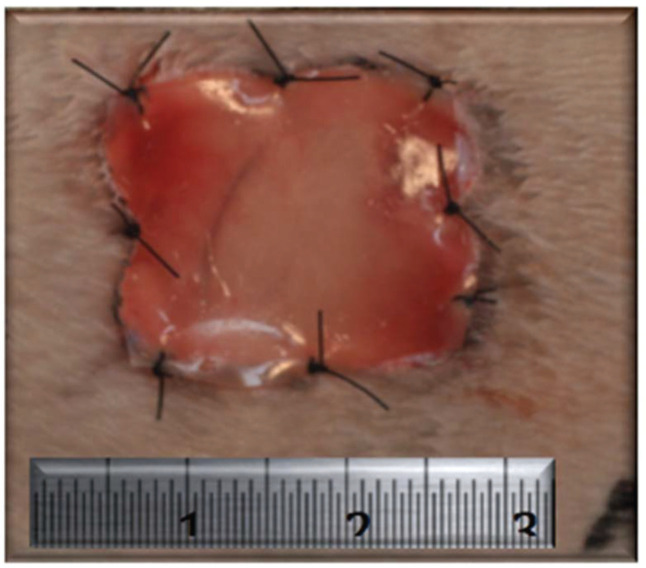
Dermal matrix attached to the wounds in the matrix group.

After the surgical procedures were performed as described above, no intervention was performed in the wounds of the animals in the CG, and healing by secondary intention was observed. In the MG, the wounds of the animals were covered with ADM (Integra^®^, Integra Life Sciences Corporation, Plainsboro, NJ, USA). For each lesion, a 400-mm^2^ square fragment of ADM was transferred to the recipient bed and fixed to the tissue margin with 4.0 nylon sutures distributed at the midpoints of the four sides and at the four angles for a total of eight fixation points (Figure 2). An occlusive dressing was applied using a transparent Tegaderm adhesive patch (3M, St. Paul, MN, USA) in both groups. During the immediate postoperative period, the animals received an analgesic subcutaneously (tramadol - 2 mg/kg single dose) and an antibiotic intramuscularly (enrofloxacin - 5-mg/kg single dose).

After 3, 14 and 21 days, a lesion sample with a 2.0-mm margin was collected from all rabbits by complete excision of the wound, and the samples were sent for histological analysis. The first sample, taken at 3 days, was collected from the wound on the right side of the animal; the second, taken at 14 days, was collected from the cranial wound on the left side of the animal; and the third, taken at 21 days, was collected from the caudal wound on the left side of the animal.

No anesthetic or surgical complications were observed during the study. The skin wounds healed without any symptoms or signs of infection. No animals were lost due to other causes.

### Calculation of the wound healing rate

A photographic record of wound healing over 21 days was taken by a blinded evaluator. The photos were of the left caudal wound in all animals. All images were taken with a millimeter ruler placed next to the wound. The images were processed in ImageJ software (NIH, Bethesda, MD, USA) to measure the wound area.

To calculate the wound healing rate, the following formula was used:

(initial wound area - wound area on the day/initial wound area) × 100

### Histology

Light microscopy (Nikon Ti-U, Nikon Instruments, Inc., NY, USA) was used for histological and histomorphometric analyses with hematoxylin and eosin (H&E) staining. Type I and type III collagen were quantified by staining with picrosirius red (Sirius Red F3BA, Sigma-Aldrich Corp., St. Louis, MO, USA). The tissue stained with picrosirius red was analyzed by polarized light microscopy (Nikon E-800, Nikon Instruments Inc., NY, USA) for the presence of type I and type III collagen fibers. An equal area was quantified in all slides and was obtained by scanning the image for 10 fields at 40× magnification.

The areas corresponding to each polarization were summed per slide, and the density of each type of polarization was calculated in relation to the total area studied. The analysis was performed using ImageJ software version 4.5 (NIH, Bethesda, MD, USA), and the results are expressed as the mean density of the two different types of collagen fibers.

### Histomorphometry

Images of the slides were obtained with a high-resolution AxioCam camera (Carl Zeiss, GmbH, Oberkochen, Germany) coupled to a Zeiss microscope. AxioVision Rel 4.8 software was used to measure skin thickness.

Five epidermal measurements of each specimen were taken of the area bounded by the lower surface of the basal layer and the outer surface of the granular layer in different sections of the same slide. The same procedure was used to determine dermal thickness. The upper limit was the lower surface of the basal layer, and the lower limit was the subcutaneous adipose tissue.

All histological measurements were performed independently by two independent observers who were blinded to group assignment, and the mean value was considered for the analysis.

The biopsied portion selected for microscopic analysis was the margin of the wound exactly at the limit between healthy skin and the wound.

### Statistical analysis

To compare the groups, one-factor analysis of variance (ANOVA) models were used, followed by Tukey’s multiple comparison test or the Mann-Whitney test to determine differences. The level of significance was set to 0.05. The statistical software IBM SPSS for Windows, version 22.0 (Armonk, NY: IBM Corp.) was used.

## RESULTS

### Calculation of wound contraction

**Figure 3:**
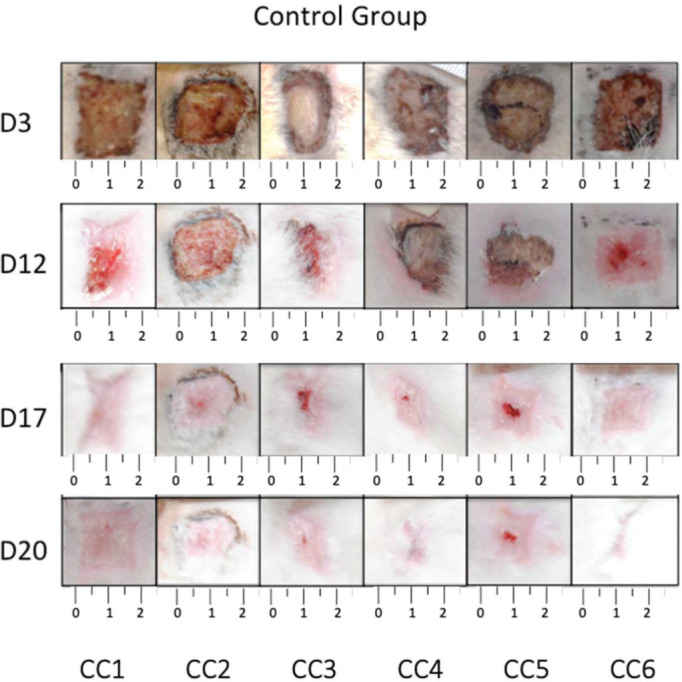
Photographic record of wound healing for each rabbit in the control group.

**Figure 4:**
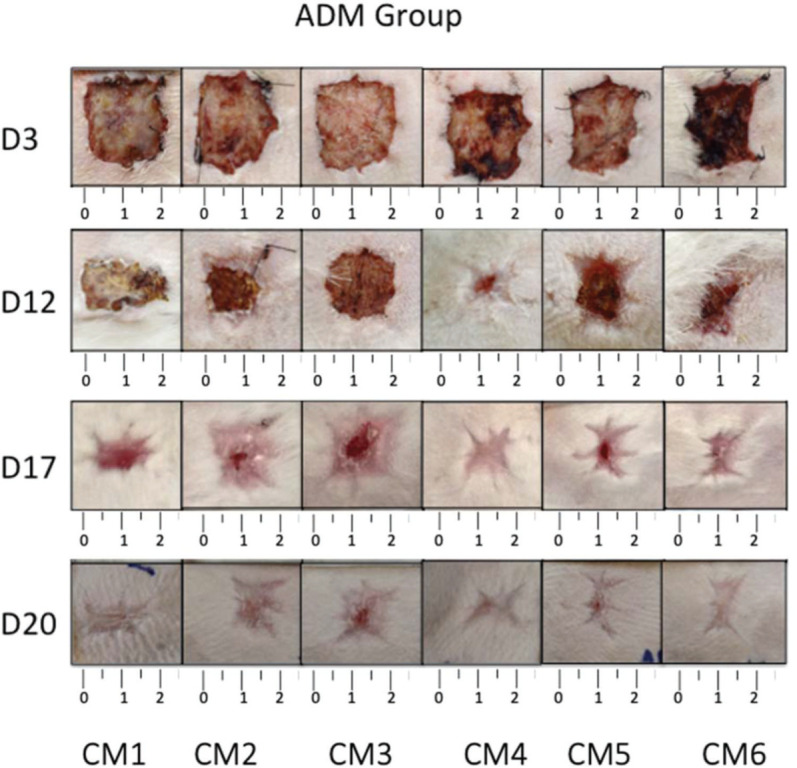
Photographic record of wound healing for each rabbit in the matrix group.

**Figure 5:**
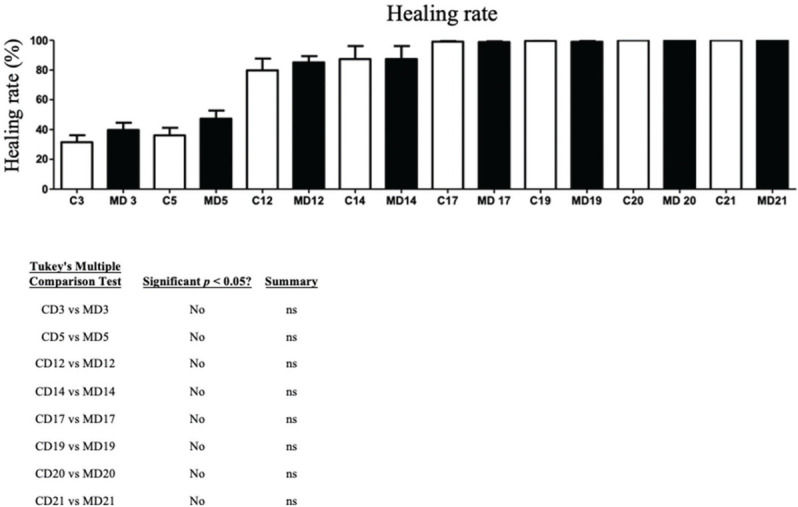
Wound healing rate in both groups. Cx (rabbits from the control group on day x); MDx (rabbits from the matrix group on day x).

The wounds on the animals’ back were photographed daily (Figures 3 and 4), and the percentage of wound contraction was recorded (Figure 5). In both groups, complete wound healing occurred in 20 days, and there was no significant difference at any timepoint during the evaluation (*p*>0.05).

### Histomorphometry

#### Epidermis

**Figure 6:**
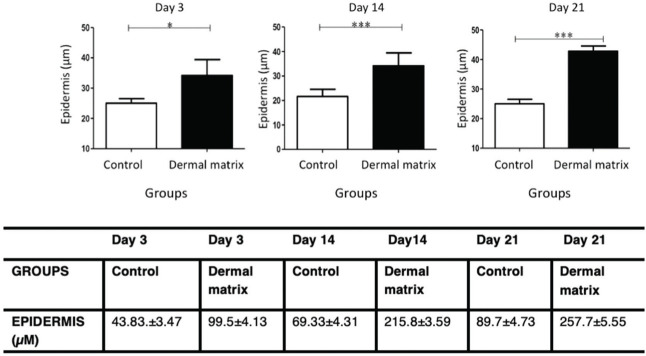
Epidermal thickness progression in both groups. *p<0.05; ***p<0.001.

**Figure 7:**
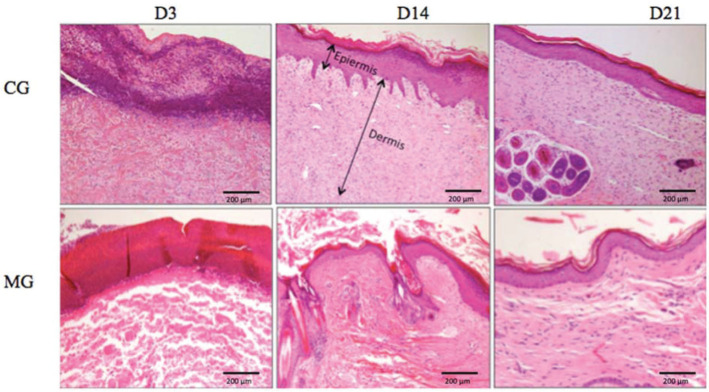
Histological photographs showing the thickness of the dermis and epidermis over time (H & E, 10×).

The MG presented greater epidermal thickness than did the CG on day 3 (*p*<0.05) and on days 14 and 21 (*p*<0.001) (Figures 6 and 7).

#### Dermis

**Figure 8:**
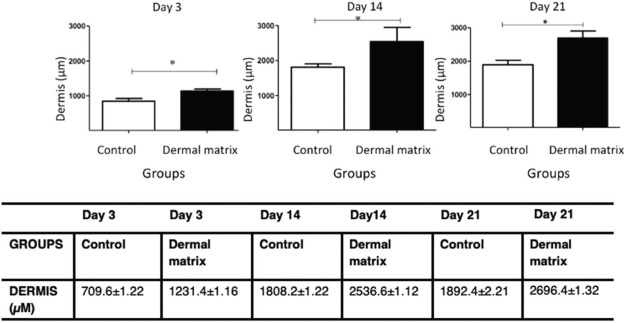
Dermal thickness progression in both groups. *p<0.05.

The MG had greater dermal thickness than did the CG throughout the evaluated period (Figures 7 and 8).

#### Collagen

**Figure 9:**
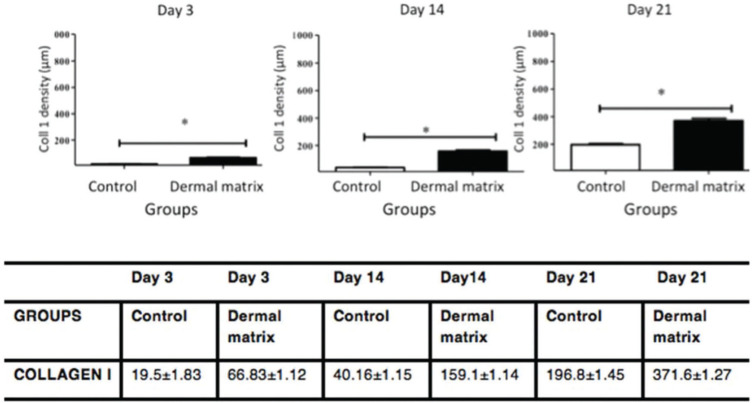
Collagen type I density progression in the CG and MG. *p<0.05.

**Figure 10:**
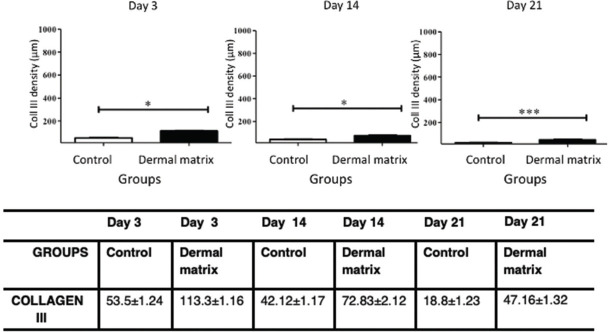
Collagen type III density progression in the CG and MG. *p<0.05 ***p<0.001.

**Figure 11:**
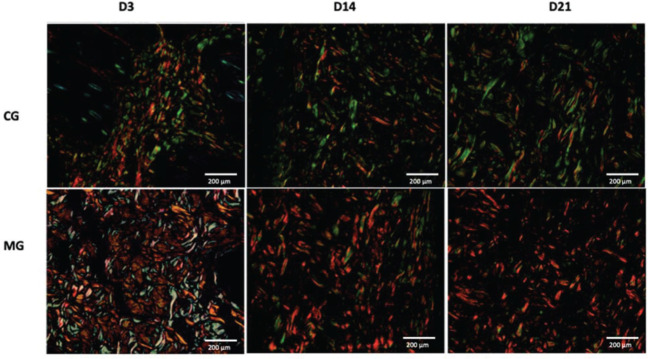
Histological photographs showing the distribution of type I (red) and type III (green) collagen fibers during the healing process in both groups (picrosirius red, 10×).

The MG presented higher collagen type I density than did the CG (*p*<0.05) throughout the study period (Figures 9 and 11). Type III collagen levels, despite the decrease throughout the study, always remained higher in the MG (Figures 10 and 11).

## DISCUSSION

Skin tissue engineering is an important adjuvant in the treatment of skin wounds. In the triad of cells, scaffold and regulatory factors, scaffolds have been the subject of much research and major advances (17,21). However, even with recent advances, scientific evidence from experimental studies on the effects and functions of matrices in the healing process is of fundamental importance for expanding the clinical indications of different dermal matrices.

Biocompatible matrices can simulate the extracellular matrix of native tissue, providing a porous structure and an environment favorable to cell growth, proliferation and differentiation, which are partly responsible for wound healing (22).

The selection of the number and position of the lesions followed the model established by Cross et al. (24). The number of biopsies is justified for the use of as few animals as possible, and the selected days were determined to follow the progression of wound healing. The three evaluation periods correspond to important milestones in the healing process; day 3 is the beginning of neovascularization, day 14 is the peak of collagen production, and stabilization between collagenogenesis and collagenolysis occurs on day 21 (39).

If we consider different locations for the wounds, randomization may lead to samples that are not comparable at different times and healing phases. Therefore, the sequence of analysis was defined and replicated in all animals in both groups to avoid any bias.

In the present study, the effect of ADM was evaluated macroscopically using the wound healing rate, which was not significantly different with or without the use of ADM throughout the study period. These results confirm the findings of other studies that also showed no difference in healing time in skin wounds when ADM alone was used (17,23). Rabbits have a healing process in which wound contraction is predominantly mediated by myofibroblasts (24). Thus, the action of ADM in the proliferative phase of healing is due to fibroproliferation more than wound contraction (19).

Wound healing occurs through two main mechanisms: wound contraction and re-epithelialization. Wound healing follows a centripetal pattern, i.e., from the margin of the wound to the center, and epithelialization begins within 48 hours; therefore, samples were taken from the wound margin. In animals considered prey in nature, such as rabbits and rats, the healing process occurs predominantly by contraction rather than re-epithelialization; in contrast, in humans, re-epithelialization is the main mechanism. Thus, the fact that the healing rate showed no significant difference may be explained by the predominance of the wound contraction mechanism in this type of animal, which may have outweighed any effect caused by the presence of ADM in the lesion. However, the presence of ADM caused an increase in the epidermal and dermal thickness throughout the evaluation period.

Although there was no difference in wound healing time, the quality of the scar tissue was higher in the MG. Histomorphometry of the lesions showed a significant increase in epidermal thickness in the MG compared to that in the CG throughout the study period. Several studies using ADM on skin wounds have found more pronounced proliferation of blood vessels and fibroblasts within ADM (25-27). The blood vessels that proliferate inside the ADM supply nutrients and oxygen for epidermal cell maintenance and proliferation (28). In addition, in the literature, the viability and functionality of the constituent elements of the dermis are well documented to be crucial for the viability and functionality of epidermal cells (29,30). ADM behaves as an appropriate substrate for the adhesion, growth and differentiation of keratinocytes (31,32). The facilitating role of ADM in the migration and proliferation of keratinocytes in skin wounds is confirmed by increasing the gene expression of Keratin 19 (K19) and ß-1 integrin, which are the biomarkers of stem cells of keratinocytes (33). A thickened epidermis is directly related to a thickened dermis, and an increase in the constituent elements of the dermis is fundamental for keratinocyte proliferation.

In addition to the increase in epidermal thickness, the dermis also showed increased thickness with the use of ADM. ADM is a biodegradable dermal scaffold that is replaced by new tissue at the recipient site and is part of the matrix integrated into the skin. The collagen dermal replacement layer serves as a facilitating structure for the infiltration of macrophages, lymphocytes, blood vessels and, mainly, fibroblasts into the wound bed (7). As healing occurs, an endogenous collagen matrix is deposited by fibroblasts, and simultaneously, the artificial dermal layer is degraded (4). Although the dermal matrix undergoes a decellularization process, its recognition as a foreign body by the organism cannot be overlooked (34). Thus, the immune system will be activated, leading to an increase in proinflammatory cytokines and several growth factors such as FGFb (basic fibroblast growth factor) (27). The sum of these factors may explain the increased dermal thickness observed in the MG and demonstrates the advantage of using ADM for the treatment of complex wounds.

In healthy skin, the ratio between collagen I/III is 3.5:1. Type I collagen is responsible for the tensile strength, continuity and protective function of the skin. During the skin remodeling and maturation process, it is expected that type III collagen decreases in relation to type I collagen to revert to the ratio found in normal skin (19). In addition, the decrease in type I collagen and the increase in type III collagen are associated with thinner and more flexible collagen fibers, leading to reduced tensile strength (28). The greater amount of type I and III collagen in the wounds in which ADM was used demonstrates an overall increase in collagen in the scar tissue. The density of type III collagen in the MG decreased over time but always remained higher than that in the CG. Type I collagen exhibited a progressive increase in density, which was always higher in the MG than in the CG. These findings corroborate the results of previous studies that also found an increase in total collagen in skin wounds in the presence of ADM (29) and may once again demonstrate the benefits of the clinical use of ADM for the treatment of complex wounds.

Although ADM is associated with low vascularization of the recipient bed, in the experimental models in this study, the recipient beds of the wounds in both the MG and CG did not show impaired vascularization since the models were healthy rabbits without any induction of microangiopathy, as in diabetic rabbit models. Therefore, similar to other preclinical studies involving cutaneous healing (35-37), the aim of this study was not to evaluate and quantify the number of blood vessels but to analyze the influence of ADM in an environment with good vascularization.

In a recent clinical study, objective and subjective improvements in scar quality were demonstrated using the Vancouver Scar Scale (VSS) and the Patient and Observer Scar Assessment Scale (POSAS) in a group that received ADM combined with a skin graft compared to the control group in which only a graft was used. Studies such as this reinforce the hypothesis that the increase in collagen and in dermal and epidermal thickness caused by the presence of ADM improves scar quality, which is a clinically relevant factor for patients (38).

New adjuvant factors such as growth factors, vascular stromal fraction, plasma proteins and stem cells are being studied to optimize the use of ADMs, allowing the expansion of their clinical use. The aim is to improve the quality of scars not only by increasing the thickness of the dermis, epidermis and collagen but also by increasing vascularization in order to accelerate healing, which would have a major impact in clinical practice.

## CONCLUSION

The use of ADM increased the thickness of the dermis and epidermis and the amount of type I and III collagen in healing skin wounds and did not alter the wound contraction rate.

## AUTHOR CONTRIBUTIONS

Carvalho-Júnior JC performed the surgeries on the rabbits, analyzed the histological images and wrote the manuscript. Zanata F and Aloise AC participated in the rabbit surgeries and were responsible for the manuscript review. Ferreira LM conceived the original idea, provided orientation during research development and was responsible for the manuscript review.
